# Three-Blind Validation Strategy of Deep Learning Models for Image Segmentation

**DOI:** 10.3390/jimaging11050170

**Published:** 2025-05-21

**Authors:** Andrés Larroza, Francisco Javier Pérez-Benito, Raquel Tendero, Juan Carlos Perez-Cortes, Marta Román, Rafael Llobet

**Affiliations:** 1 Instituto Tecnológico de la Informática, Universitat Politècnica de València, Camino de Vera, s/n, 46022 València, Spain; alarroza@iti.es (A.L.); fjperez@iti.es (F.J.P.-B.); rtendero@iti.es (R.T.); jcperez@iti.es (J.C.P.-C.); 2 Department of Epidemiology and Evaluation, IMIM (Hospital del Mar Research Institute), Passeig Marítim 25-29, 08003 Barcelona, Spain; mroman@hmar.cat

**Keywords:** deep learning, image segmentation, mammography

## Abstract

Image segmentation plays a central role in computer vision applications such as medical imaging, industrial inspection, and environmental monitoring. However, evaluating segmentation performance can be particularly challenging when ground truth is not clearly defined, as is often the case in tasks involving subjective interpretation. These challenges are amplified by inter- and intra-observer variability, which complicates the use of human annotations as a reliable reference. To address this, we propose a novel validation framework—referred to as the three-blind validation strategy—that enables rigorous assessment of segmentation models in contexts where subjectivity and label variability are significant. The core idea is to have a third independent expert, blind to the labeler identities, assess a shuffled set of segmentations produced by multiple human annotators and/or automated models. This allows for the unbiased evaluation of model performance and helps uncover patterns of disagreement that may indicate systematic issues with either human or machine annotations. The primary objective of this study is to introduce and demonstrate this validation strategy as a generalizable framework for robust model evaluation in subjective segmentation tasks. We illustrate its practical implementation in a mammography use case involving dense tissue segmentation while emphasizing its potential applicability to a broad range of segmentation scenarios.

## 1. Introduction

Image segmentation, the process of partitioning an image into meaningful regions, is a critical task in computer vision. By identifying and delineating objects or regions of interest, segmentation serves as a foundational step in numerous applications. In industrial settings, it is used for quality control, defect detection, and autonomous navigation, enabling robots to identify and manipulate objects on assembly lines [1]. In the medical domain, segmentation aids in the analysis of anatomical structures, the identification of pathologies, and treatment planning, such as delineating tumors for radiotherapy [2]. Environmental applications, including land-use mapping and disaster management, also rely heavily on accurate segmentation [3]. These examples highlight the importance of segmentation across diverse domains, underscoring its role in advancing technological and societal goals.

Despite its utility, image segmentation faces significant challenges. Inter- and intra-observer variability pose problems, especially in domains requiring subjective interpretation, such as medical imaging. In radiology, differences in training, experience, and judgment can lead to inconsistent annotations, affecting diagnoses and treatment plans [4]. Similarly, in industrial applications, variability in manual quality assessments can result in inefficiencies or the misclassification of defects [5]. This variability underscores the need for standardized methodologies and robust automated systems to enhance reliability in segmentation tasks across all application areas.

Addressing these challenges requires the extensive validation of segmentation algorithms. Deep learning models, particularly those designed for segmentation, need rigorous evaluation to ensure their robustness and generalizability across diverse datasets [6]. A comprehensive validation framework includes performance metrics, such as precision and recall, and assessments of the model’s ability to handle variations in data quality, resolution, and noise [7]. These systematic validation efforts are crucial to bridging the gap between algorithmic performance in controlled experimental settings and real-world deployment, ultimately improving the trust in and adoption of these models.

A specific example of these challenges can be found in mammography, where the accurate assessment of breast dense tissue is critical. Breast density, assessed from digital mammograms, is a known biomarker related to a higher risk of developing breast cancer. Its precise quantification is essential for improving cancer detection and screening effectiveness. Therefore, precise segmentation of dense tissue in mammograms is crucial for enhancing diagnostic accuracy. Recent advancements in segmentation techniques, particularly those leveraging deep learning, have demonstrated enhanced capabilities in distinguishing between dense and non-dense tissues, thereby facilitating more accurate breast cancer assessments [8].

Building on these advancements, this study introduces a three-blind validation strategy for deep learning-based segmentation. While dense tissue segmentation in mammography serves as a use case, the proposed approach is applicable to various segmentation tasks across different domains. A key challenge in evaluating deep learning-based segmentation models is the inherent subjectivity of the task, leading to intra- and inter-observer variability. Since no perfectly defined ground truth exists, discrepancies between the model and human experts cannot always be attributed to errors in one or the other. This validation strategy provides a structured way to assess agreement between labelers and the model, offering insights that can support the refinement of human annotations and the improvement of automatic segmentation models.

## 2. Materials and Methods

### 2.1. Validation Strategy

**Figure 1:**
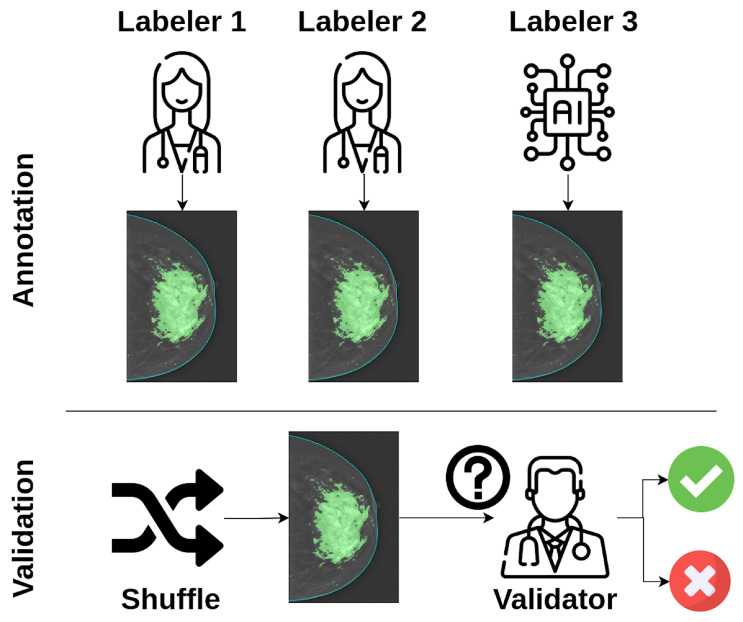
Workflow of the three-blind validation strategy for image segmentation. Multiple human labelers and deep learning models annotate a defined number of images. The annotations are then shuffled to anonymize their source, and an independent validator reviews them to ensure an impartial assessment of their quality. While the figure illustrates two human labelers and one deep learning model, the strategy can be extended to any number of human or automatic annotators. This figure has been designed using resources from flaticon.com (accessed on 13 December 2024).

Ensuring reliable, high-quality segmentations is essential for developing and evaluating deep learning models for image segmentation. To address this, we propose a three-blind validation strategy, as illustrated in Figure 1. This approach compares the performance of deep learning models with human specialists by anonymizing annotation sources, ensuring unbiased evaluation by an expert validator. A predefined set of images is annotated by multiple human labelers and deep learning models. The annotations are then shuffled to remove identifying information about their origin. An independent validator reviews these shuffled annotations, providing an impartial assessment of their quality.

This strategy enables the analysis of inter-observer variability by comparing annotations from different human labelers and the model. Additionally, intra-observer variability can be assessed by having the validator unknowingly review a subset of segmentations twice at random. By ensuring that these repeated segmentations are presented to the validator without their awareness of the repetition, this approach provides assessment of the validator’s consistency.

### 2.2. Validation Tool

A custom interactive tool was developed to support the three-blind validation strategy by enabling a third specialist (validator) to independently assess segmentation masks. Built using the Python-based Streamlit library [9], the tool provides a user-friendly interface where the validator is presented with one segmentation mask at a time, without any indication of its origin (human or model). The tool randomly determines the order of presentation for segmentations from different sources, ensuring blinding.

For each displayed segmentation, the validator selects one of four predefined categories: correct, oversegmented, undersegmented, or incorrect. The tool also includes a comment box for optional qualitative feedback. This structure supports the standardized quantitative and qualitative evaluation of segmentations while preserving the independence of the assessment.

**Figure 2:**
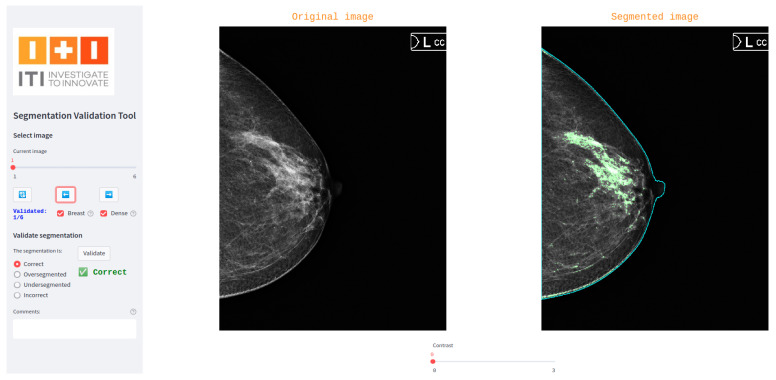
Screenshot of the tool used for three-blind validation in the breast dense tissue segmentation use case.

Figure 2 shows a screenshot of the interface used in our breast dense tissue segmentation use case. In this version, the interface was tailored to display digital mammograms and associated masks. A generalized version of the tool has also been developed and made publicly available via GitLab (https://egitlab.iti.es/praia-salud/segmentation-validation-tool.git, (accessed on 1 May 2025)). This version is designed to be compatible with any segmentation task, as it can be easily configured for different label categories and image modalities.

### 2.3. Use Case: Breast Dense Tissue Segmentation

The objective of this use case is to exhaustively validate a model for segmenting dense tissue in digital mammograms. The main challenges include variations in images from different acquisition devices and inter- and intra-reader variability [10]. Variability in human annotations is a critical consideration in this use case. Inter-observer variability refers to differences in annotations between different radiologists, while intra-observer variability reflects the consistency of annotations made by the same radiologist at different times. These sources of variability complicate the evaluation of model performance, as discrepancies may arise not only from model errors but also from human subjectivity. As such, our validation strategy incorporates analyses to assess both inter- and intra-observer agreement as described in the analyses presented below.

#### 2.3.1. Dataset

**Figure 3:**
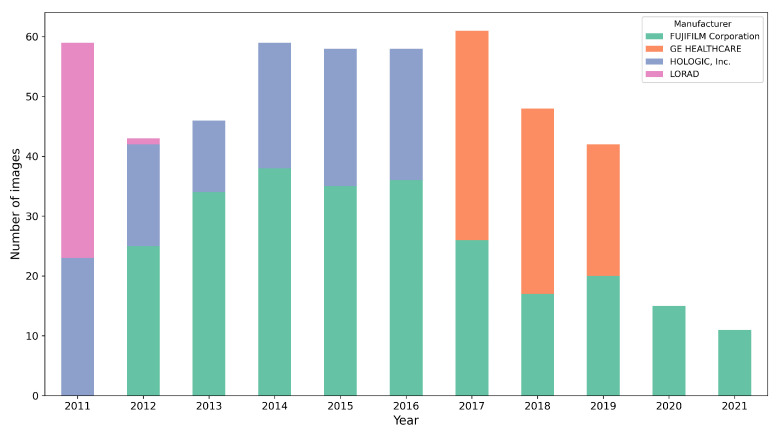
Distribution of the 500 mammograms according the year and acquisition device.

We utilized a dataset comprising 500 studies obtained from the Hospital del Mar Research Institute (IMIM). This dataset was exclusively extracted for the three-blind validation presented here. It consists of mammograms from four different acquisition devices, collected over a period of 10 years (2011–2021). To simplify the procedure, only craniocaudal (CC) views were used. All the mammograms are of the type *for presentation.* Figure 3 illustrates the distribution of mammograms by year and acquisition device, highlighting a well-balanced representation across five distinct devices over the covered period. This diverse composition emphasizes the dataset’s robustness and suitability for validating segmentation performance.

#### 2.3.2. Deep Learning Model

**Figure 4:**
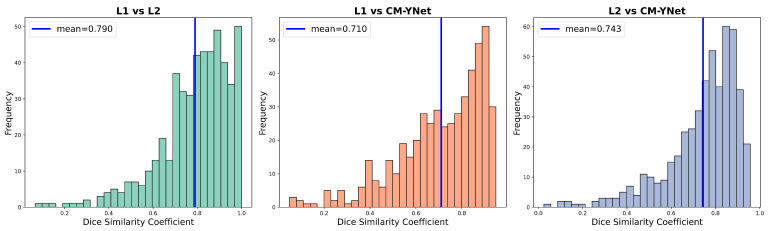
Distribution of DSC values between the labelers and the output generated by the CM-YNet model on 500 validation craniocaudal (CC) images.

The CM-YNet is a deep learning model developed by our group that automatically segments the dense tissue in digital mammograms. For full architectural and training details of CM-YNet, including the model’s design rationale and evaluation against expert annotations, readers are referred to Larroza et al. [8]. Our previous results indicate that the CM-YNet model performs well, achieving a Dice Similarity Coefficient (DSC) comparable to that obtained between two specialists. This suggests that radiologists tend to agree more with the CM-YNet segmentation than with each other. Table 1 and Figure 4 show the DSC values obtained for the 500 validation images compared with the expert labelers. Given the well-known variability among expert readers, the next step is to verify that a third specialist agrees with CM-YNet as much as with the other two specialists, introducing the concept of three-blind validation strategy outlined in Section 2.1.

#### 2.3.3. Data Annotation

Data annotation was performed with an in-house developed tool named Futura Breast, which can be used to interactively segment the dense tissue using two parameters: the brightness corrector α and the fibroglandular tissue threshold $th_{F}$. These parameters guide the segmentation process as described in detail in Larroza et al. [8]. Two expert radiologists (L1 and L2) were instructed to perform data annotation with this tool. In total, each expert independently annotated 500 validation CC images. The same 500 images were also segmented by the automatic CM-YNet model. Therefore, we obtained a total of 1500 segmentations that were used for the three-blind validation. Additionally, 300 randomly selected images (100 from L1, 100 from L2, and 100 from CM-YNet) were included twice. This random sample allowed for the calculation of intra-observer variability for the specialist conducting the validation. In total, the third specialist (validator) randomly validated 1800 segmentations.

The use of two human annotators was a pragmatic choice, balancing the need for reliable expert annotations with the high cost and time demands of manual labeling in clinical imaging. Recruiting two board-certified radiologists was considered the minimum acceptable setup to ensure diversity in expert opinion while maintaining feasibility within project constraints.

We conducted the three-blind validation twice using two independent radiologists with experience in breast imaging, referred to as Validator 1 (V1) and Validator 2 (V2). These validators were not involved in the initial annotations and had no prior knowledge of the segmentation sources (L1, L2, or CM-YNet). The first validation was performed by V1, and the second validation was carried out by V2. For the second validation, the breast delineation method was improved using the approach presented in [11]. Additionally, based on lessons learned from the first validation, V2 received refined instructions on the label definitions to improve consistency and reduce potential discrepancies. The specific results and observations from both validation rounds will be described in the Results Section.

#### 2.3.4. Evaluation Metrics

To assess the validation results, we employed a range of commonly used evaluation metrics. We provide brief definitions and contexts for each metric used.

- Dice Similarity Coefficient (DSC): The DSC is a spatial overlap index widely used to evaluate segmentation tasks. It quantifies the similarity between two sets by computing the overlap relative to their combined size. It can take values ranging from 0 to 1, with a higher value indicating a higher similarity [12].
- Accuracy: Accuracy is defined as the proportion of correctly classified instances among the total number of instances. While it provides a general sense of performance, it can be misleading in imbalanced datasets [13].
- Cohen’s Kappa: Cohen’s Kappa measures the agreement between two raters while accounting for agreement occurring by chance. It is especially useful when comparing annotations from different sources or observers [14].
- Balanced Accuracy: Balanced Accuracy accounts for imbalanced class distributions by averaging the recall obtained on each class. It is computed as the average of sensitivity (recall) and specificity [15].
- F1 Score: The F1 score is the harmonic mean of precision and recall, providing a single metric that balances both. It is particularly useful when the dataset has class imbalance and when false positives and false negatives carry different costs [13].
- Precision: Precision measures the proportion of true positive predictions among all positive predictions, reflecting the model’s ability to avoid false positives [13].
- Recall: Recall, also known as sensitivity, measures the proportion of true positives among all actual positives, capturing the model’s ability to detect relevant instances [13].

## 3. Results

### 3.1. First Validation

The validation tool provided four labels for assessing segmentations: correct, incorrect, oversegmented, and undersegmented. The incorrect label was originally intended for cases where the segmentation was entirely wrong and could not be classified as either oversegmented or undersegmented. However, after reviewing the 1800 segmentations, validator V1 reported that he used the incorrect label only rarely and did not consistently apply a clear criterion for distinguishing between oversegmented, undersegmented, and incorrect. For example, some cases involving the segmentation of the pectoral muscle as dense tissue were labeled interchangeably as either oversegmented or incorrect. Consequently, to evaluate the results of V1, we decided to simplify the classification by grouping the labels into two broader categories: agreement and disagreement. This issue was resolved for the second validation, for which a clearer validation criterion was established in advance.

#### 3.1.1. Agreement with Each Labeler

**Figure 5:**
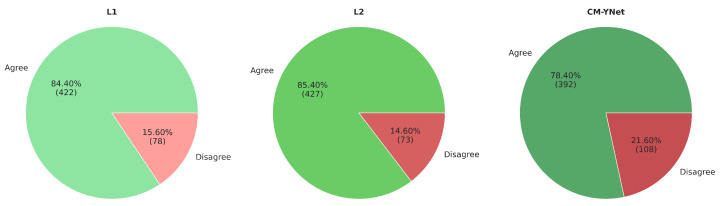
Agreement percentages between V1 and each evaluated labeler for a total of 1500 segmentations.

Figure 5 presents the agreement percentages for each evaluated labeler (L1, L2, and CM-YNet). The agreement with manual segmentations (L1 and L2) is higher than that with the automatic segmentation (CM-YNet). These percentages are based on a total of 1500 segmentations.

**Figure 6:**
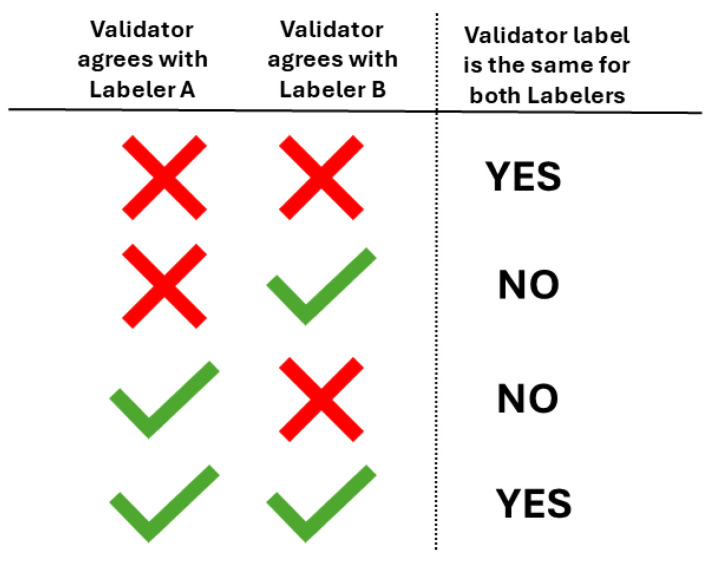
Given two labelers, which in our case can be any combination of L1, L2, or CM-YNet, we indicate if the validator marked the same label (agreement or disagreement) for both labelers.

Subsequently, we analyzed the DSC between labelers, based on V1’s agreement or disagreement with each segmentation presented. For each validated image, we indicate whether the validator’s agreement or disagreement with two of the labelers was the same or not, as illustrated in Figure 6.

The DSC values are presented in Table 2. It is noteworthy that the DSC is higher in cases where V1 had the same label on two given segmentations, which corresponds to the highest percentage of images across all cases.

#### 3.1.2. Intra-Observer Variability

**Figure 7:**
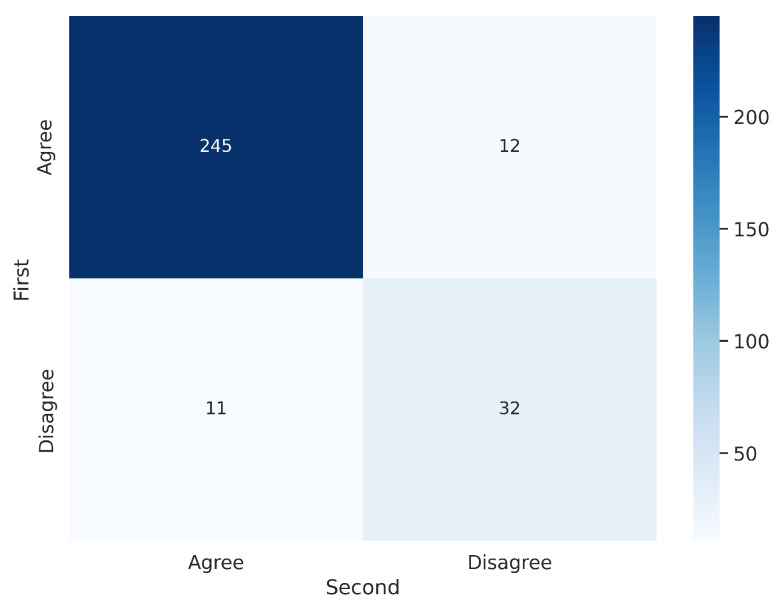
Confusion matrix for the 300 segmentations shown twice to V1. V1 exhibited inconsistency in 23 out of 300 cases (7.67%).

**Figure 8:**
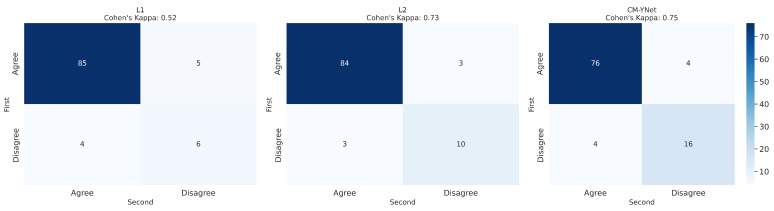
Confusion matrices per labeler for the 300 segmentations shown twice to V1.

To analyze intra-observer variability, the 300 segmentations that were randomly presented to V1 twice were used. Figure 7 and Figure 8 illustrate the confusion matrices for V1’s first and second evaluations of these segmentations. The results indicate that, in most cases, V1 demonstrated consistency in his decisions. Table 3 summarizes the corresponding metrics.

**Figure 9:**
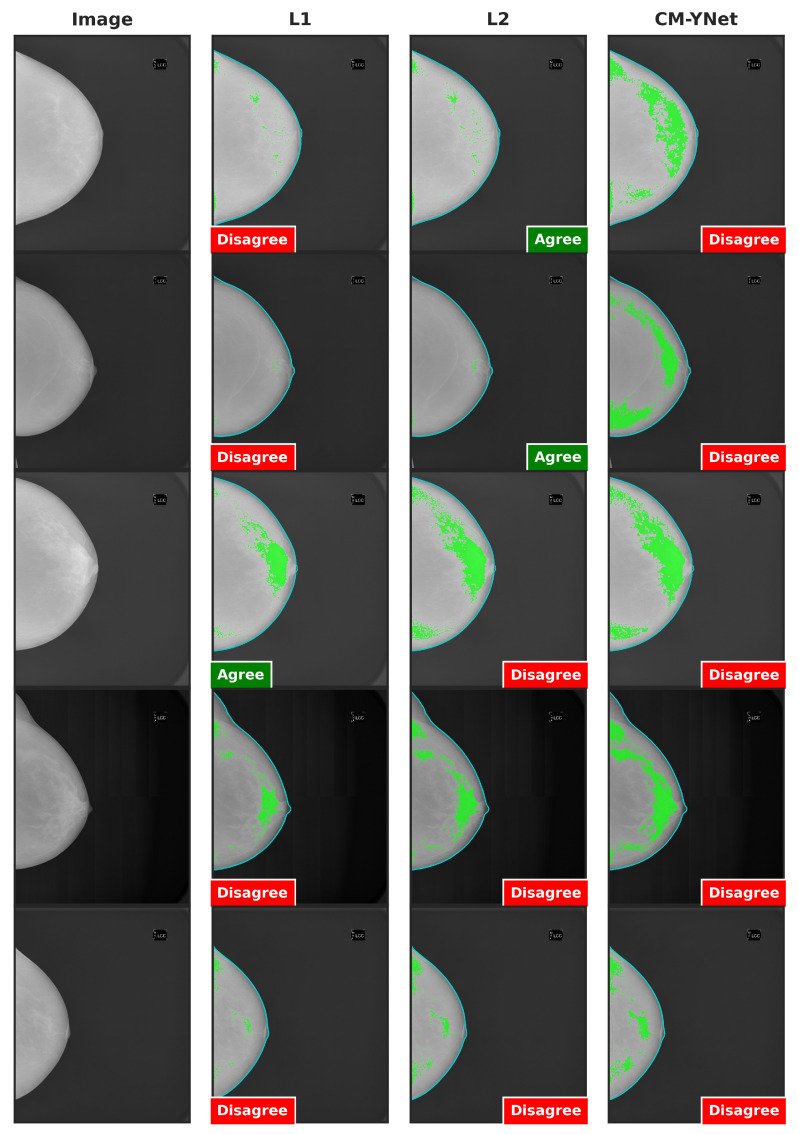
Examples of segmentations that were shown twice to V1 to analyze the intra-observer variability. In these examples, V1 indicated disagreement with CM-YNet on both occasions.

Figure 9 presents examples of images segmented by CM-YNet where V1 disagreed with the automatic segmentation in both evaluations of the same segmentation. This disagreement was observed in 16 out of 300 images, as depicted in Figure 8. Notably, these images were acquired using older devices (HOLOGIC and LORAD in Figure 3), which could explain the increased difficulty in accurately segmenting these lower-quality images, even for expert annotators.

Further analysis of the intra-observer confusion matrices (Figure 5), which reflect the consistency of each validator’s repeated assessments using binary labels (Agree vs. Disagree), reveals that the segmentations originally labeled by L1 exhibit the lowest intra-observer agreement. This indicates that the validator (V1) was less consistent when evaluating L1’s segmentations, potentially due to greater variability or ambiguity in those masks. In contrast, CM-YNet’s segmentations, although not always labeled as correct, were assessed more consistently by V1 across repeated validations. These results suggest that consistency in evaluation may not always align with accuracy and that model-generated masks can elicit more reproducible judgments even if they are more frequently judged as incorrect.

#### 3.1.3. Exploring the Causes of Disagreement

For a given image, three segmentations were available: one each by L1, L2, and CM-YNet. These were presented to V1 in random order. Thus, the order of appearance (first, second, or third) refers to whether the segmentation from a particular labeler was the first, second, or third version of the same image seen by V1. This order may influence the validator’s judgment, as familiarity with the image increases with repeated exposure.

**Figure 10:**
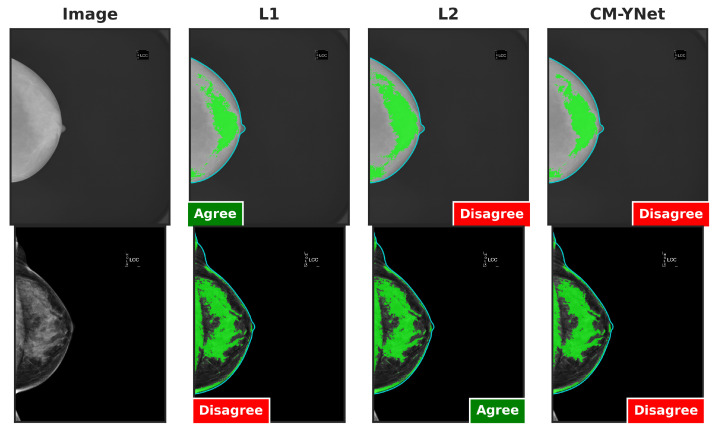
Examples of segmentations where V1 changed his opinion over time: from agree to disagree (first row), and from disagree to agree (second row). Although the visual layout follows a fixed column order (original image, L1, L2, and CM-YNet), the validator viewed the segmentations in a different sequence. For the first-row example, the presentation order was L1, L2, CM-YNet; for the second-row example, it was L1, CM-YNet, L2.

Figure 10 shows two examples where V1’s decisions changed depending on the order in which the segmentations were presented to them during the validation process, even though the segmentations from the three labelers were visually similar. Importantly, the order discussed here refers to the sequence in which the segmentations appeared to V1, not the fixed column order used in the figure layout. In the first-row example, the segmentation by L1 was shown first to V1, who marked it as agreement; however, V1 later marked disagreement for the same image when it reappeared with segmentations by L2 and CM-YNet. In the second-row example, V1 initially marked disagreement for the first two segmentations they saw (L1 and CM-YNet) but later marked agreement for the third (L2).

These observations were corroborated by V1, who was consulted about these specific examples without indicating him the order of appearance:

- Example 1: “In the inner quadrants of the three images something that is not dense tissue is segmented, so they would be *oversegmented*, but they also do not include all the glandular tissue of the breast, so they would also be *undersegmented*. We could consider them incorrect. At some point, I probably concluded that the machine or the labelers could not avoid including something from the inner quadrants without sacrificing the fibroglandular tissue, and that is why I marked the first one as *correct* It would be good to know in what order I read them”.
- Example 2: “The three images seem to be oversegmented. It may be that I marked L2 as *correct* because I evaluated it last and understood that it was difficult not to include the pectoralis major since the dense tissue was so well delineated in the segmentation. In this case, it would be good to know in what order I read the three images”.

It is worth mentioning that the segmentations in these examples are very similar (high DSC). Additionally, as V1 mentioned for the second example, the pectoral muscle appears segmented as dense tissue. This issue arose in several images. For all images, the breast delineation was automatically annotated with a threshold-based method implemented in Futura Breast. That implementation removes the pectoral muscle only on mediolateral oblique (MLO) views where the pectoral muscle is more likely to appear. All the images evaluated in this study are craniocaudal (CC) views, and in these, the implemented algorithm was unable to remove the pectoral muscle.

**Figure 11:**
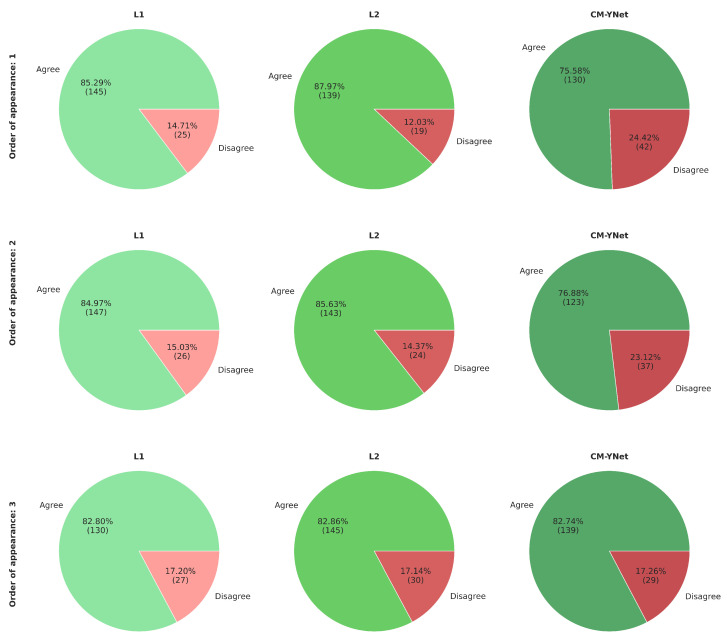
Agreement percentage between V1 and each labeler according to the order of appearance of the segmentations. The first row shows the results when the corresponding segmentations were the first to appear for a given image, the second row shows the segmentations that appeared second for a given image, and similarly for the third row.

Considering V1’s comments and seeing how the order of appearance of the segmentations influenced V1’s decision, we reanalyze the agreement percentages but taking into account the order of appearance (Figure 11). Our analysis shows that the agreement rate with CM-YNet increased with later appearances: from 75.6% when CM-YNet’s segmentation appeared first, to 82.7% when it appeared third. This suggests that V1 developed a more refined labeling criterion after seeing other versions of the same image. Interestingly, for L1 and L2, we observed the opposite trend: agreement with V1 slightly decreased as their segmentations appeared later in the sequence. This may indicate that V1’s exposure to alternative segmentations led to increased scrutiny or preference for different delineation styles, particularly when viewing human-labeled segmentations after a model output or another human’s annotation. These findings highlight that the sequential context in which segmentations are presented can influence expert validation outcomes.

According to the results of the first validation and V1’s descriptions, CM-YNet tends to oversegment. This oversegmentation is most likely due to the inclusion of the pectoral muscle, as mentioned earlier. For this reason, we performed a second validation with a different validator (V2), first improving the pectoral muscle exclusion and also providing clearer instructions on how to use the validation labels consistently over time.

### 3.2. Second Validation

For the second validation, we first improved the breast delineation to effectively remove the pectoral muscle in CC images. The new breast delineation method implemented is described in our previous work [11]. With this method, we ensured that the dense segmentation performed by CM-YNet no longer included the pectoral muscle, preventing bias for V2. Additionally, as seen in the first validation, we aimed to ensure that V2 did not change his criteria over time. To this end, we provided clearer instructions on how to consistently use the validation tool labels:

- Use the correct label only if the segmentation matches the dense tissue, not based on assumptions about the limitations of the labelers’ methods.
- Use the oversegmented/undersegmented labels only when it is evident that the segmentation includes significantly more or less tissue than the actual dense tissue.
- Use the incorrect label only for rare cases, such as when the pectoral muscle is included as dense tissue.

#### 3.2.1. Agreement with Each Labeler

**Figure 12:**
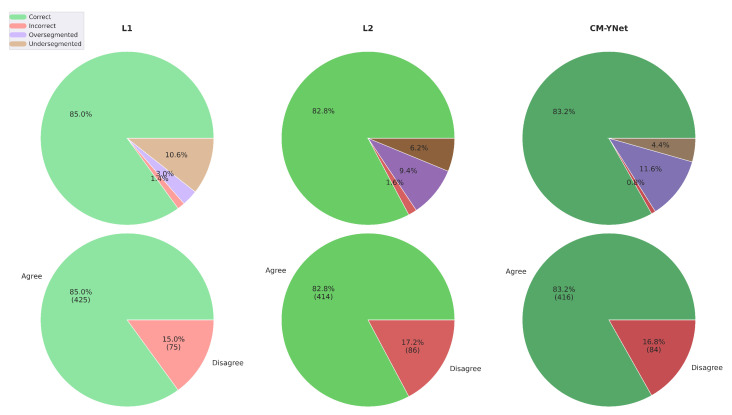
Agreement percentage between V2 and each evaluated labeler for a total of 1500 segmentations.

Figure 12 illustrates the agreement percentages between V2 and each evaluated labeler (L1, L2, and CM-YNet). The results indicate that the agreement percentage is comparable between manual segmentations—L1 (85.0%) and L2 (82.8%)—and the automatic segmentation CM-YNet (83.2%). This percentage accounts for all segmentations (1500 in total). Notably, the primary source of disagreement varies across labelers: undersegmentation is the main cause of disagreement with L1, whereas oversegmentation is predominant with L2 and CM-YNet. These findings highlight a higher level of agreement between V2 and the automatic segmentation compared to the results from the first validation (Figure 5).

The DSC values in Table 4 are based on V2’s labels when evaluating pairs of segmentations for the same image, as described in Figure 6. In accordance with the findings from the first validation, the DSC values are higher in cases where V2 assigned the same label to both segmentations. These cases correspond to the majority of images across all evaluated labelers.

#### 3.2.2. Intra-Observer Variability

**Figure 13:**
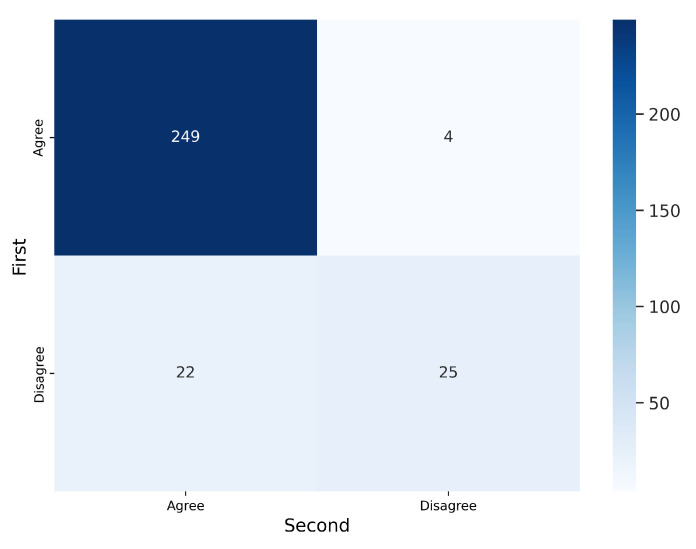
Confusion matrix for the 300 segmentations presented twice to V2. Inconsistencies were observed in 26/300 cases (8.67%).

**Figure 14:**
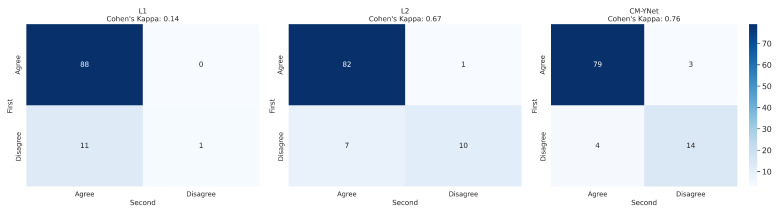
Confusion matrices for each labeler, based on the 300 segmentations shown twice to V2.

As in the first validation, we analyzed intra-observer variability by randomly presenting 300 segmentations (100 per labeler) to V2 twice. Figure 13 and Figure 14 display the confusion matrices for V2’s first and second evaluations of these segmentations. The results indicate that V2 was consistent in the majority of cases but less consistent than V1. Table 5 summarizes the corresponding metrics.

**Figure 15:**
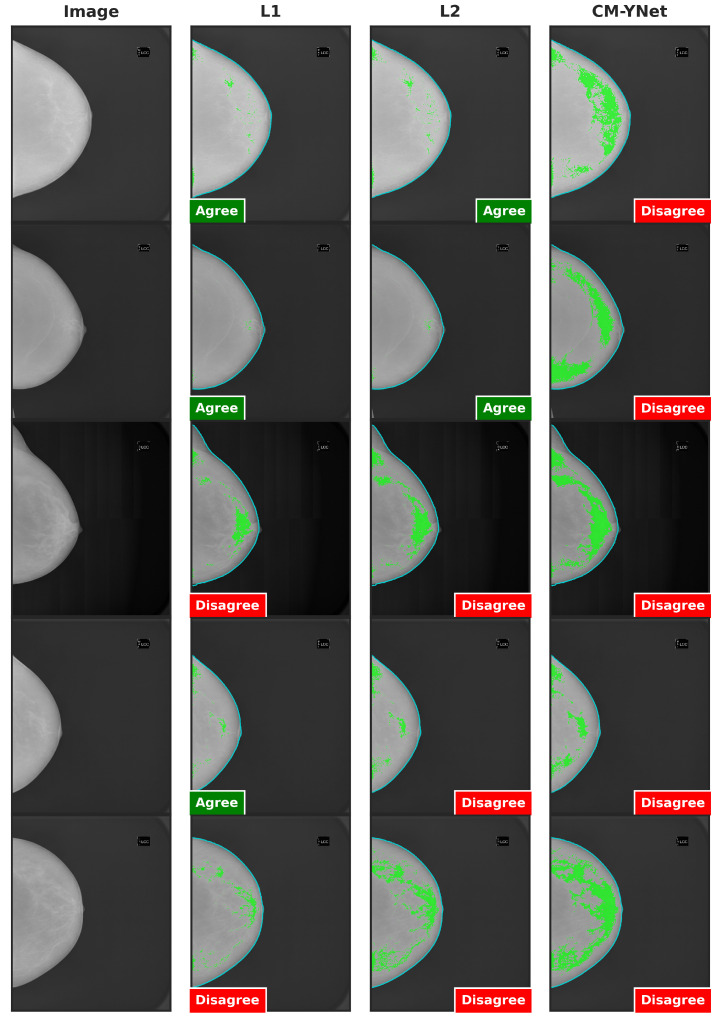
Examples of segmentations that were shown twice to V2 to analyze intra-observer variability. In these examples, V2 indicated disagreement with CM-YNet on both occasions.

Figure 15 provides examples of segmentations produced by CM-YNet where V2 expressed disagreement both times the same segmentation was shown. These disagreements occurred in a total of 14 images. Specifically, V2 assigned 12 cases as oversegmented and 2 cases as undersegmented.

#### 3.2.3. Exploring the Causes of Disagreement

**Figure 16:**
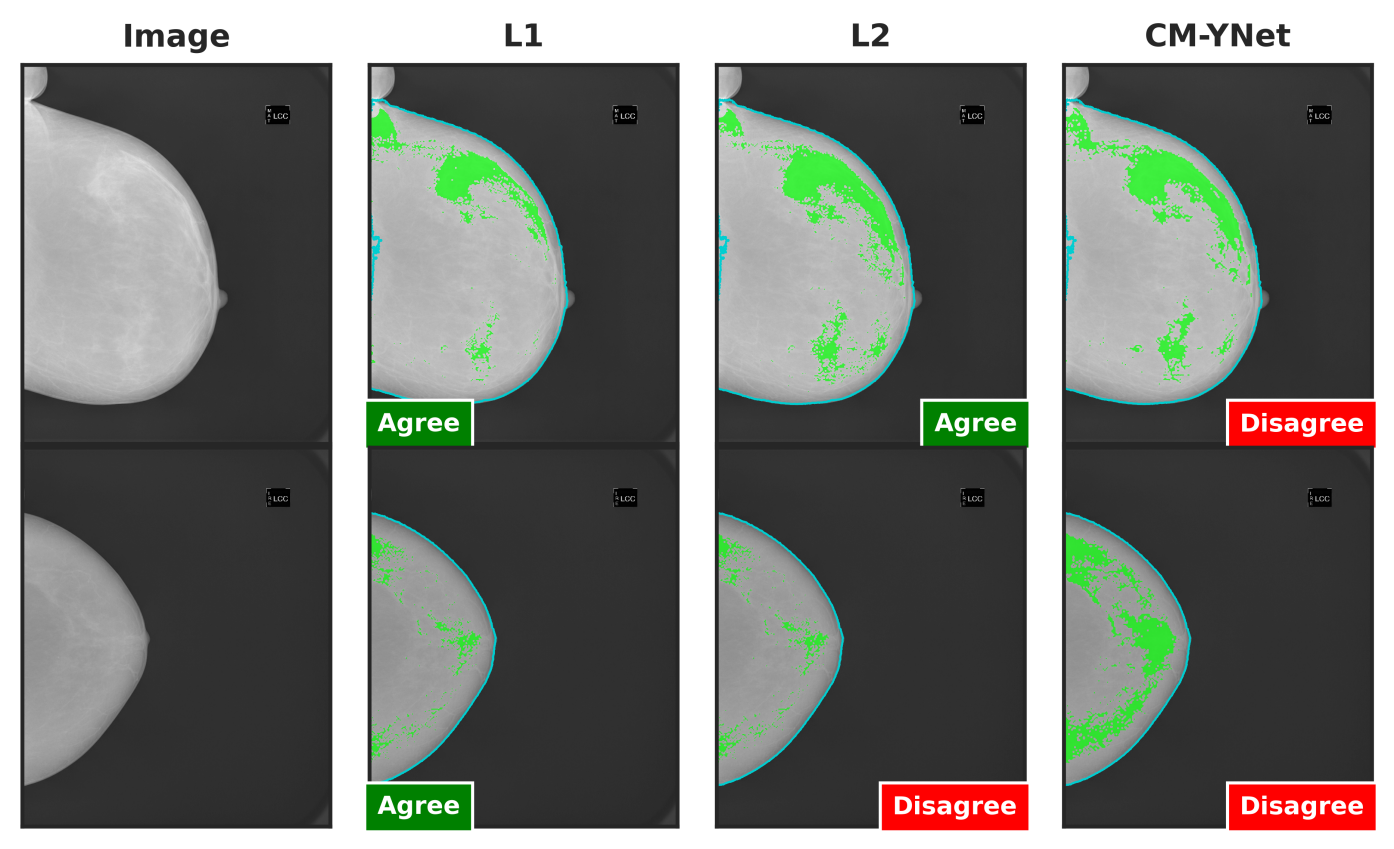
Examples of segmentations where V2 assigned different labels despite identical segmentations (DSC = 1): L2 and CM-YNet in the first row, and L1 and L2 in the second row. Although the visual layout follows a fixed column order (original image, L1, L2, and CM-YNet), the validator viewed the segmentations in a different sequence. In the first-row example, the order of appearance was L1, CM-YNet, and L2. In the second-row example, the order was CM-YNet, L1, and L2.

Figure 16 illustrates two examples where V2’s decisions varied depending on the order in which the segmentations were presented. In the example in the first row, V2 initially marked the first segmentation (L1) as correct. However, when the next two segmentations (CM-YNet and L2) were shown, both with identical segmentations (DSC = 1), V2 changed his decision to oversegmented for CM-YNet and then correct again for L2. In the example in the second row, V2 indicated the first segmentation (CM-YNet) as versegmented but later marked L1 as correct and L2 as oversegmented. Similar to the first example, L1 and L2 had identical segmentations (DSC = 1).

**Figure 17:**
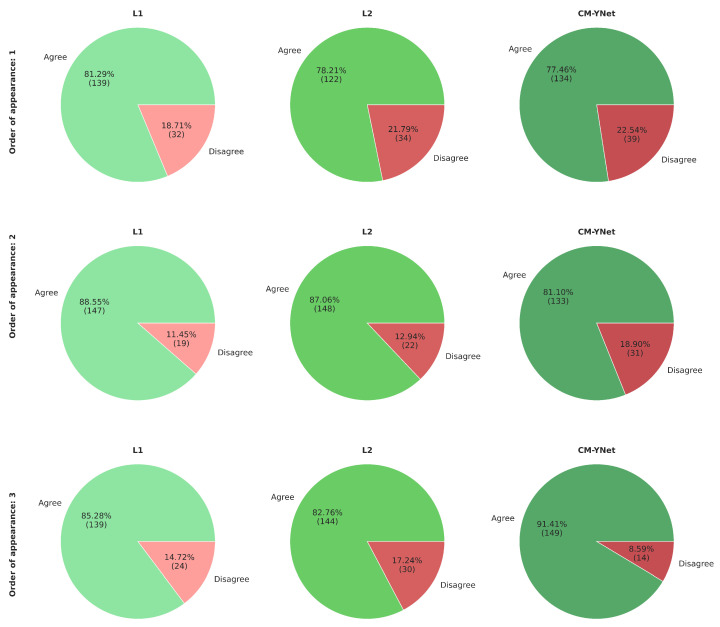
Agreement percentages between V2 and each labeler based on the order in which segmentations were presented. The first row shows the results when the segmentations were presented first for a given image. The second and third rows correspond to cases where the segmentations appeared second and third, respectively.

Figure 17 presents the agreement percentages between V2 and each labeler as a function of their order of appearance. Notably, the agreement with CM-YNet improved significantly, rising from 77.46% when CM-YNet was presented first to 91.41% when it appeared last. This upward trend in agreement was not observed for L1 and L2. For these labelers, agreement percentages increased when they appeared second then decreased again when they were shown third, showing no clear pattern. Therefore, even though the intra-observer variability indicates more inconsistency for V2 compared to V1, it appears that V2 maintained a consistent criterion over time. The inconsistencies found were more likely due to the inherent intra-observer variability that is well-known for this type of segmentation tasks, especially in the medical domain.

**Figure 18:**
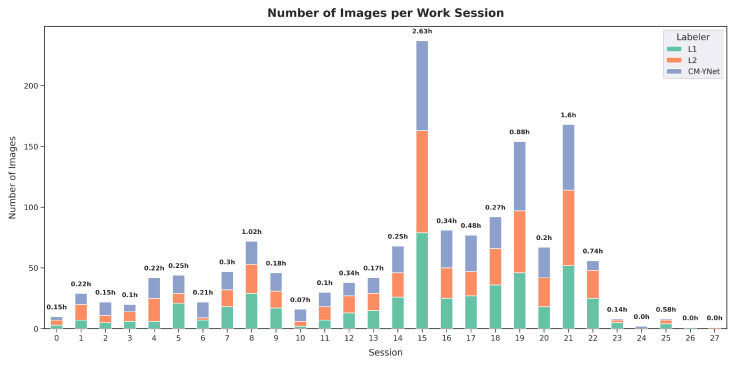
Summary of V2’s labeling sessions, showing the duration and number of images labeled in each session.

Finally, we wanted to explore whether the evaluation sessions influenced V2’s results. To this end, an analysis of the validation sessions was conducted. A session was defined as a continuous period during which V2 reviewed images without taking extended breaks. A break of more than one hour marked the start of a new session. A total of 28 sessions were identified (Figure 18), with the longest session lasting nearly three hours. Notably, the final two sessions each consisted of labeling only one image, with a duration of approximately one minute.

**Figure 19:**
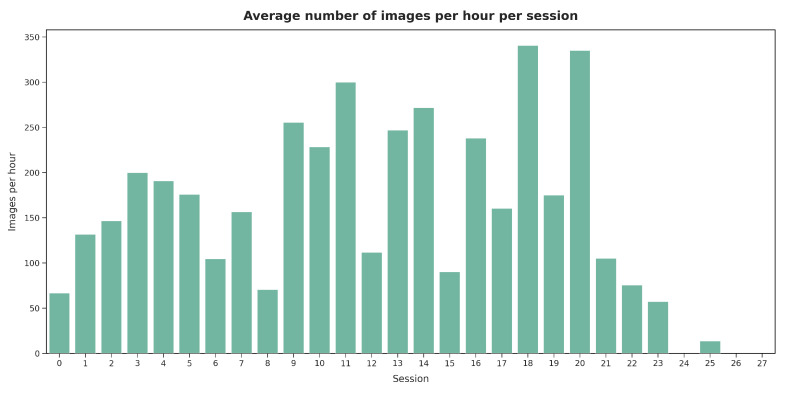
Average number of images labeled per hour during each session by V2.

**Figure 20:**
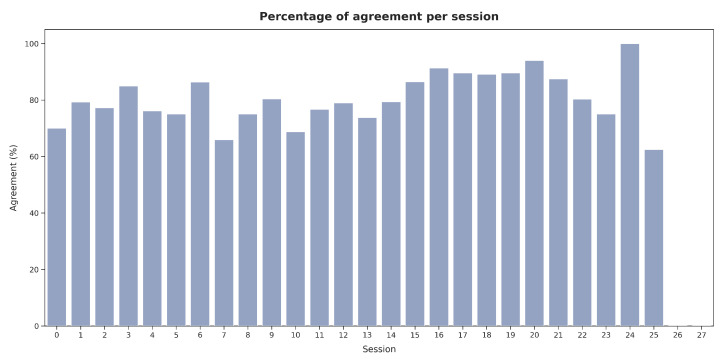
Agreement percentage with the presented segmentations across sessions for V2.

Figure 19 presents the average number of images labeled per hour across sessions. Shorter sessions showed a tendency toward the faster validation of images, while in longer sessions (e.g., sessions 15 and 21), V2 spent more time evaluating each image. However, as illustrated in Figure 20, no significant relationship was observed between the labeling speed in different sessions and the agreement percentage with the presented segmentations.

## 4. Discussion

In this study, we proposed a three-blind validation strategy to compare the agreement between a validator and three different labelers (two human and one deep-learning-based segmentation model). This approach was demonstrated using breast dense tissue segmentation, where a third validator independently evaluated the segmented images without prior knowledge of their origin. Since there is no absolute ground truth—due to inherent intra- and inter-observer variability among human labelers—this strategy provides insight into the level of similarity between the labelers, as assessed by the validator. This can help detect errors in the labels, which can then be refined by human reviewers or used to improve the automatic segmentation model.

The first validation highlighted a critical issue in the preprocessing step of our automatic segmentation model. Specifically, the breast delineation algorithm used prior to dense tissue segmentation failed to exclude the pectoral muscle in CC mammograms, resulting in its incorrect classification as dense tissue by the CM-YNet model. Addressing this limitation, we refined the breast delineation method and subsequently conducted a second validation with a different specialist. For this second validation, we provided clearer and more detailed instructions to the second validator (V2) to ensure consistent use of the validation tool’s labels. This improvement led to a higher agreement between V2 and both the automatic and manual segmentations, compared to the results obtained in the first validation.

A critical step toward improving model performance is the identification of systematic errors. In this study, errors in the first validation were detected through expert feedback and visual inspection of the validation results. However, it is important to note that both the nature of these errors and the appropriate corrective strategies can vary significantly depending on the specific use case, dataset composition, and segmentation objectives. As such, error analysis and subsequent model refinement should be tailored to the application context rather than relying on a one-size-fits-all approach.

Unlike traditional validation schemes that focus solely on metric-based comparisons to reference labels, our approach engages an independent validator in a blinded setting to assess the degree of agreement between human and automated segmentations. This design aligns with recent calls for more robust and unbiased evaluation methods in subjective annotation contexts [16,17]. While human-in-the-loop strategies have been used to improve annotation quality and model performance [18], few works have employed a blinded evaluation of multiple sources simultaneously, making our approach a unique contribution to the validation literature.

### 4.1. Limitations

Despite its strengths, the proposed validation strategy has several limitations. First, the presented use case involved only two radiologists and one independent validator. While this provided a manageable and controlled comparison, it may not capture the full range of inter-observer variability, which is known to influence perceived model accuracy in medical imaging [19,20]. Increasing the number of annotators and applying aggregation methods such as STAPLE [21] could reduce individual bias and improve the reliability of the assessment.

Second, although the validator was blinded to the source of the segmentations, the lack of clinical context during evaluation may reduce validity in real-world diagnostic scenarios [16]. Third, the validation framework currently functions as a static benchmarking tool and does not support real-time model refinement. Iterative improvement mechanisms—such as those used in active learning or semi-supervised frameworks [22,23]—could further enhance the practical utility of this strategy.

Finally, while our results were demonstrated on mammographic segmentation, the generalizability of this framework to other domains (e.g., industrial inspection or environmental monitoring) remains to be empirically validated. As such, claims regarding its broad applicability should be interpreted with caution until further validation studies are conducted.

### 4.2. Future Work

Building on these findings, several future directions can enhance the utility and scope of the three-blind validation framework. First, clinical validation studies are needed to assess how well this strategy translates into real-world diagnostic workflows and whether it can support or augment radiologist decision-making. Incorporating richer forms of validator feedback and integrating domain-specific context may also improve the robustness of the assessment.

Second, we plan to apply the three-blind validation approach across diverse segmentation tasks—such as those found in industrial inspection and other areas of medical imaging—to evaluate its scalability and generalizability in domains where ground truth is inherently uncertain or contested.

## 5. Conclusions

This study presented a three-blind validation strategy to compare the agreement between human labelers and a deep-learning-based segmentation model. Applied to breast dense tissue segmentation, this strategy helped identify and address issues in the automatic model while highlighting the potential of automatic segmentation for reproducible results. By ensuring an unbiased evaluation, it offers valuable insights that can help improve segmentation models and support their broader adoption in medical imaging and beyond.

## Figures and Tables

**Table 1:** DSC values obtained by the CM-YNet model compared against the expert labelers on the 500 validation images.

L1 vs. L2	L1 vs. CM-YNet	L2 vs. CM-YNet	Closest vs. CM-YNet
0.790±0.160	0.710±0.187	0.743±0.162	0.773 ± 0.157

The Closest vs. CM-YNet Dice Similarity Coefficient (DSC) is computed by selecting, for each individual image, the higher DSC value between L1 vs. CM-YNet and L2 vs. CM-YNet. The reported value is the average of these per-image maxima across the 500 validation images.

**Table 2:** DSC values for the different labelers (L1, L2, and CM-YNet), based on the of labels assigned by V1 to the evaluated segmentations.

Labelers	V1 Label Is the Same	DSC	95% CI
L1 vs. L2	No (55)	0.636±0.215	(0.578,0.694)
	Yes (445)	0.809±0.141	(0.796,0.822)
L1 vs. CM-YNET	No (78)	0.614±0.225	(0.563,0.664)
	Yes (422)	0.728±0.174	(0.712,0.745)
L2 vs. CM-YNET	No (73)	0.648±0.202	(0.601,0.695)
	Yes (427)	0.760±0.149	(0.746,0.774)

**Table 3:** Metrics derived from the confusion matrix based on the 300 images reviewed twice by V1.

Accuracy	Acc. 95% CI−	Acc. 95% CI+	Kappa	Balanced Accuracy	F1	Precision	Recall
0.923	0.888	0.948	0.691	0.849	0.955	0.957	0.953

**Table 4:** DSC values for the different labelers (L1, L2, and CM-YNet), based on the labels assigned by V2 to the evaluated segmentations.

Labelers	V2 Label Is the Same	DSC	95% CI
L1 vs. L2	No (91)	0.699±0.193	(0.658,0.739)
	Yes (409)	0.810±0.145	(0.796,0.824)
L1 vs. CM-YNet	No (101)	0.547±0.215	(0.505,0.590)
	Yes (399)	0.752±0.154	(0.737,0.767)
L2 vs. CM-YNet	No (96)	0.619±0.199	(0.579,0.660)
	Yes (404)	0.773±0.137	(0.759,0.786)

**Table 5:** Metrics calculated from the confusion matrix for the 300 segmentations reviewed twice by V2.

Accuracy	Acc. 95% CI−	Acc. 95% CI+	Kappa	Balanced Accuracy	F1	Precision	Recall
0.913	0.887	0.948	0.611	0.758	0.950	0.919	0.984

## Data Availability

A generalized version of the three-blind validation tool, developed and provided by our team for use in any image segmentation task, has been made publicly available through GitLab https://egitlab.iti.es/praia-salud/segmentation-validation-tool.git, (accessed on 1 May 2025).
